# Supplementary material for: A Whole Blood Molecular Signature for Acute Myocardial Infarction
Source: Sci Rep. 2017 Sep 25;7:12268. doi: 10.1038/s41598-017-12166-0 (PMC5612952; doi:10.1038/s41598-017-12166-0)
Supplement: Supplementary file 1 — SUPPLEMENTARY MATERIAL [file 41598_2017_12166_MOESM1_ESM.pdf]

SUPPLEMENTARY MATERIAL

A Whole Blood Molecular Signature for Acute Myocardial Infarction

Evan D Muse,[1] Eric R Kramer,[1] Haiying Wang,[2] Paddy Barrett,[1] Fereshteh Parviz,[3] Mark A Novotny,[4] Roger S Lasken,[4] Timothy A Jatkoe,[2] Glenn Oliveira,[1] Hongfan Peng,[1] Jerry Lu,[5] Mark C Connelly,[3] Kurt Schilling,[6] Chandra Rao,[3] Ali Torkamani,[1] and Eric J. Topol[1*]

[1]*The Scripps Translational Science Institute, The Scripps Research Institute, Scripps Health, La Jolla, CA, USA*
[2]*Ortho Clinical Diagnostics, Raritin, NJ, USA*
[3]*Janssen Research & Development, LLC, Huntingdon Valley, PA, USA*
[4]*J. Craig Venter Institute, La Jolla, CA, USA*
[5]*Biological Dynamics, San Diego, CA, USA*
[6]*Ortho Clinical Diagnostics, Rochester, NY, USA*

*Correspondence to:
Eric J. Topol, M.D.
Scripps Translational Science Institute
3344 N. Torrey Pines Ct, Suite 300
La Jolla, CA 92037
USA
Tel: (858) 554-5708
Email: etopol@scripps.edu

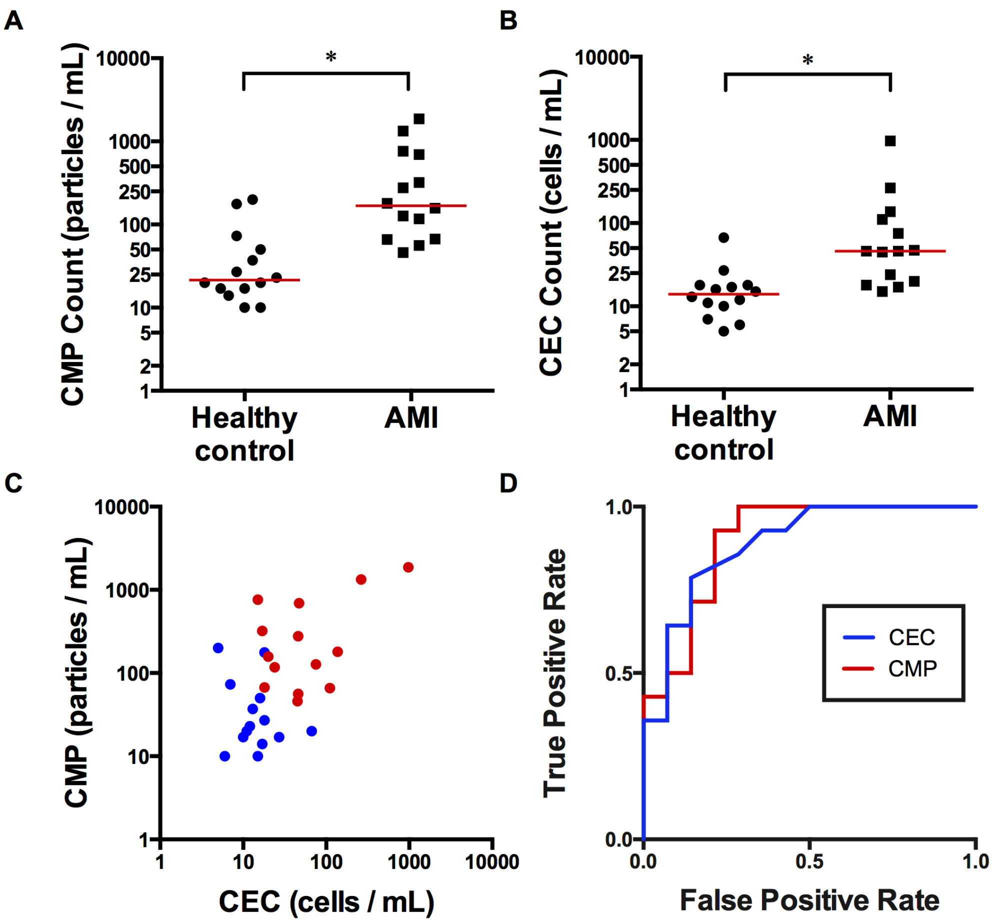

Supplementary Figure 1. Comparison of CECs and CMPs in discriminating AMI. (**A, B**) CMPs (**A**) and CECs (**B**) were enumerated in a matching subset of AMI patients (n = 14) and healthy controls (n= 14). (**C, D**) CMP and CEC counts were highly correlated (**C**, controls in blue, AMI in red) with an R-squared of 0.6922 and performed similarly by ROC-curve analysis (**D**, CMP in red, CEC in blue). * $p < 0.0002$, non-parametric Mann-Whitney two-tailed t-test.

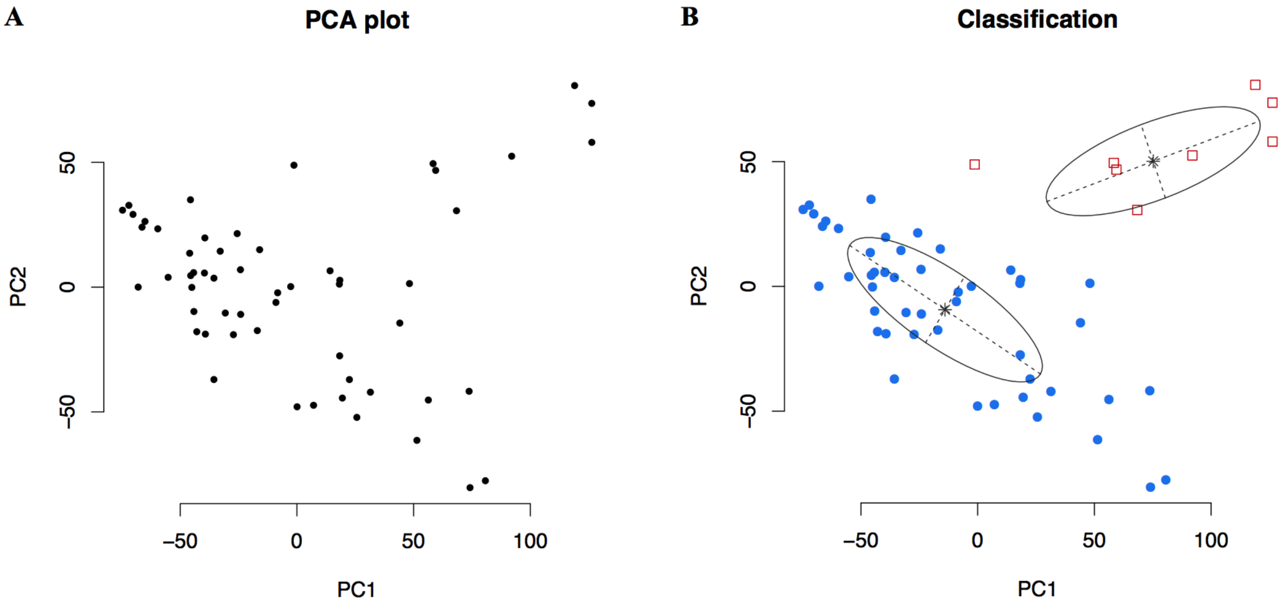

Supplementary Figure 2: Outlier detection via principle component analysis and Gaussian mixture clustering. (**A**) A scatterplot showing the first two principle components of the microarray validation set. (**B**) A Gaussian mixture clustering on the first two principle components. This clustering identifies two clusters (shown in red and blue). The samples in the red cluster (5 AMI and 3 Ctrl) were discarded as outliers.

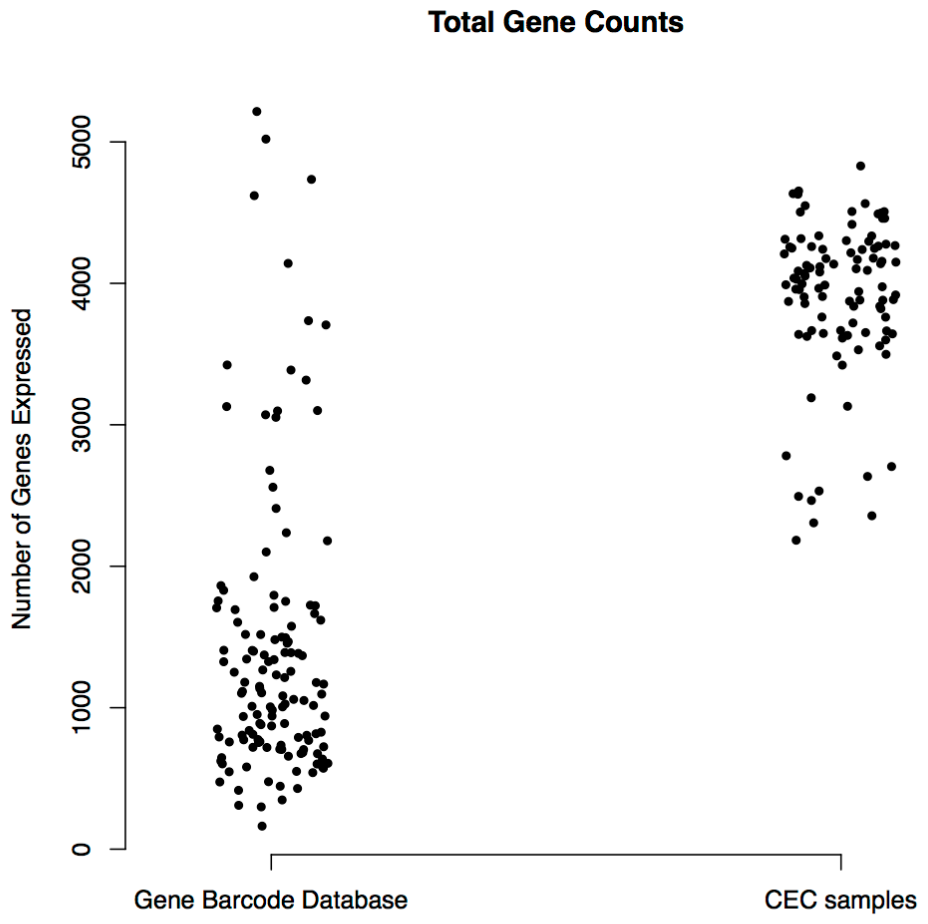

.

Supplementary Figure 3: Elevated gene counts in the enriched CECs assessed by micro array show evidence for sample heterogeneity. When compared to the gene barcode database, the total gene counts obtained from the microarray of enriched CECs are increased.

A

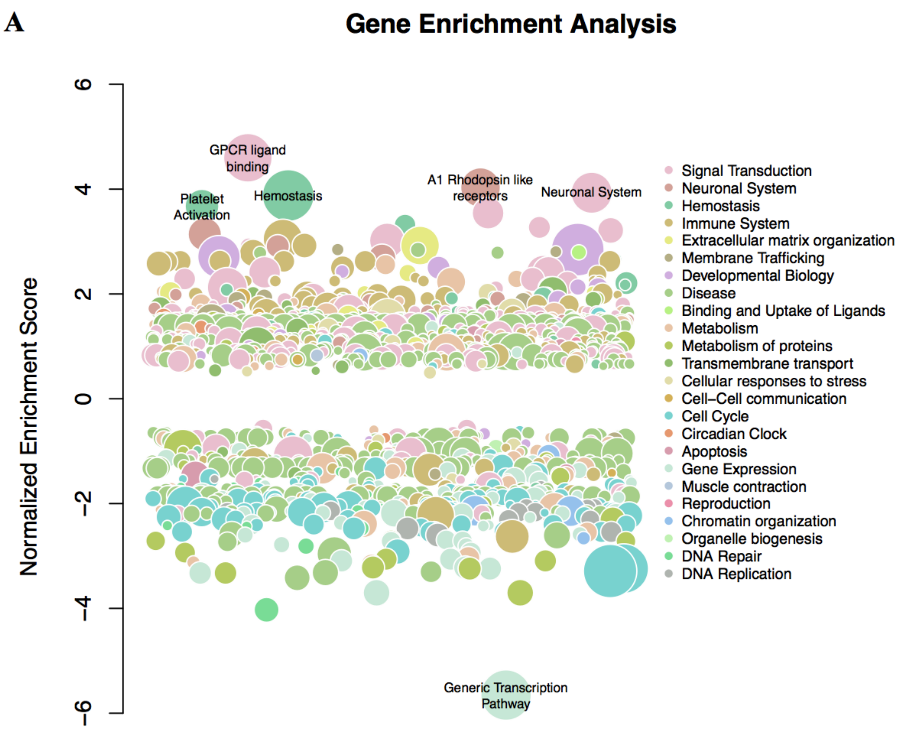

B

Upregulated in AMI

NAME	SIZE	ES	NES	NOM p-val	FDR q-val	FWER p-val
GPCR LIGAND BINDING	411	0.1975	4.5981	0	0	0
NEURONAL SYSTEM	275	0.2099	4.0292	0	0	0
CLASS A/1 (RHODOPSIN-LIKE RECEPTORS)	295	0.198	3.9309	0	0	0
HEMOSTASIS	467	0.1566	3.8785	0	0	0
PLATELET ACTIVATION, SIGNALING AND AGGREGATION	202	0.2216	3.6749	0	0	0
PEPTIDE LIGAND-BINDING RECEPTORS	178	0.2284	3.5433	0	0	0
RESPONSE TO ELEVATED PLATELET CYTOSOLIC CA2+	83	0.3108	3.3167	0	0	0
PLATELET DEGRANULATION	78	0.3166	3.2754	0	1.37E-05	1.00E-04
OLFACTORY SIGNALING PATHWAY	87	0.2991	3.2709	0	2.43E-05	2.00E-04

Downregulated in AMI

NAME	SIZE	ES	NES	NOM p-val	FDR q-val	FWER p-val
GENERIC TRANSCRIPTION PATHWAY	444	-0.232	-5.654	0	0	0
DNA REPAIR	110	-0.329	-4.026	0	0	0
METABOLISM OF RNA	245	-0.208	-3.779	0	0	0
TRANSLATION	127	-0.281	-3.701	0	0	0
INFLUENZA LIFE CYCLE	119	-0.268	-3.416	0	1.84E-05	1.00E-04
SRP-DEPENDENT COTRANSLATIONAL PROTEIN TARGETING TO MEMBRANE	92	-0.297	-3.324	0	1.53E-05	1.00E-04
INFLUENZA INFECTION	124	-0.255	-3.316	0	1.31E-05	1.00E-04
INFLUENZA VIRAL RNA TRANSCRIPTION AND REPLICATION	85	-0.302	-3.299	0	2.34E-05	2.00E-04
CELL CYCLE	497	-0.128	-3.288	0	2.08E-05	2.00E-04
CELL CYCLE, MITOTIC	428	-0.136	-3.241	0	1.88E-05	2.00E-04

Supplementary Figure 4: Gene Set Enrichment Analysis for pathways upregulated in AMI. Genes were ranked according to the fold change between AMI and controls, and a GSEA was conducted using reactome pathways as gene sets. (**A**) Each circle in the bubble represents a gene

set with the y-axis representing its normalized enrichment score and the area of the circle representing the size of the gene set. Gene sets are colored according to their reactome classification. (**B**) Several gene sets, which are related to the physiology of AMI, such as hemostasis and platelet aggregation, are significantly upregulated in AMI patients (nominal p-value of 0 represents $p < 1.0 \times 10^{-6}$).

	n (%)
Hypertension	24 (67%)
Diabetes	6 (17%)
Dyslipidemia	27 (75%)
Hypothyroidism	8 (22%)
History of Malignancy (skin, ovarian, breast)	12 (33%)
CAD	22 (61%)
Prior Stent	21 (58%)
Prior CABG	7 (19%)
Atrial Fibrillation	9 (25%)

Supplementary Table 1: Comorbidities of control population (cohort 2) used for qPCR studies.

	All AMI	Biomarker Negative AMI
**HBEGF**	0.59 (0.47-0.71)	0.50 (0.33-0.67)
**NR4A3**	0.63 (0.50-0.75)	**0.75 (0.58-0.91)**
**RNASE1**	0.76 (0.65-0.86)	0.77 (0.63-0.91)
**SYTL3**	0.74 (0.63-0.85)	**0.83 (0.71-0.95)**
**SULF1**	0.88 (0.80-0.96)	**0.93 (0.84-1.00)**
**NFKBIA**	0.68 (0.57-0.80)	**0.74 (0.60-0.88)**
**NR4A2**	0.67 (0.55-0.79)	**0.77 (0.62-0.92)**

Supplementary Table 2: Candidate gene performance in patients with non-elevated troponins. Calculated AUCs (95% CI) from ROC-curve analysis for each of the 7 individual candidate genes as assessed by qPCR from whole blood for all patients (AMI, n = 45) and patients with non-elevated troponins (AMI, n = 19).

Supplementary Methods:

Circulating microparticle (CMP) isolation and enumeration:

Whole blood in EDTA tubes from healthy control and AMI subjects was centrifuged at 1500 x g with the plasma phase separated and immediately aliquoted and frozen at -80C. Prior to CMP enumeration, 50 uL aliquots were prepared with 5x SYBR Green I double stranded DNA dye, according to labeled instructions. The sample was loaded into a flow cell and the chip was electrified using an AC function generator (Biological Dynamics, San Diego, CA) set at 7Vp-p (peak to peak), 10kHz for 10 min.[1]. An image was acquired of a ~6x6 microelectrode section of the chip (~1.2mm x 1.2mm) after 10 minutes using a fluorescent microscope with a CCD camera. A total of 100-200 uL of plasma from each patient was run (50 uL per run, 2-4 chips per sample) after which the number of particles was determined from each image. The number of particles was averaged per chip run (50 uL) and then normalized to a particle count per mL of sample.

Microarray sample preparation: Enriched CEC‑derived RNA was isolated using Trizol Reagent (Life Technologies, Carlsbad, CA) according to the manufacturer's instructions. Glycogen (Life Technologies) was added to each sample during the RNA extraction to assist in visualization of the RNA pellet. RNA was extracted from whole blood samples similarly, however without the addition of glycogen. Isolated RNA from each sample was quantified using a NanoDrop 2000 spectrophotometer (Thermo Fisher Scientific, Wilmington, DE) and Agilent Bioanalyzer 2100 (Agilent, Santa Clara, CA) according to the manufacturer's instructions and stored at −80˚C until later use. Labeled target antisense RNA (cRNA) and double stranded cDNA using the Ovation™ RNA Amplification System V2 (NuGEN, San Carlos, CA) was prepared from enriched CEC RNA samples. Purified cDNA underwent a two-step fragmentation and

labeling process using the Encore Biotin Module (NuGEN). The amplified cDNA targets were hybridized to Affymetrix human U133 Plus 2.0 array following protocols as suggested by the supplier (Affymetrix, Santa Clara, CA). Following hybridization, arrays were washed and stained using standard Affymetrix procedures before scanning on the Affymetrix GeneChip Scanner from which data was extracted using the Affymetrix Expression Console. Signal intensities from each array were normalized using the robust multichip average expression measure technique.

Microarray data analysis: Three batches of quality controls were performed using Microarray Suite 5.0 software provided by Affymetrix (www.affymetrix.com) according to the manufacturer's recommendations. Normalized expression values for the microarrays were calculated using RMA normalization [2]. Quality controls were conducted with the affy and affyQCReport R packages. A Gaussian mixture clustering of the principal components of the expression data detected eight outliers (five AMI and three control), which were discarded (**Supplementary Figure 1**). We then removed probe sets that are up-regulated in inflammatory diseases. The remaining probe sets were mapped to HGNC gene symbols. If multiple probe sets mapped to the same gene symbol, the probe set with the highest inter-quartile distance was kept for further analysis. We then used the discovery set to calculate fold changes for each probe set. Probe sets with a fold change less than two were removed. We used elastic net regression and the *glmnet* package in R to build a predictive model for acute myocardial infarction using the microarray data [3]. Parameters for the elastic net were as follows: alpha of 0.5, *pmax* of 20, binomial family, and a logistic link function. The model was trained using the discovery set and then predictions were made for the independent validation set. The performance of the model on the discovery and validation sets was evaluated using receiver-operator characteristic curves and the *pROC* package in R [4]. A differential expression analysis was run on the validation and

discovery sets using the *limma* package in R. For each probe set, a linear model was trained to predict acute myocardial infarction. P-values were calculated using an empirical Bayesian method, which were adjusted using the Bonferroni correction [5]. A gene set enrichment analysis was run on the combined set of discovery and validation samples [6]. For the GSEA, each probe's log fold change was used as the ranking statistic, and the GSEA was set to the "classic" mode. All microarray data are available from the Gene Expression Omnibus database (http://www.ncbi.nlm.nih.gov/geo) under accession code GSE66360. Code for both the preprocessing of the microarray data and the model training can be found at http://github.com/TorkamaniLab/CEC

cDNA synthesis, pre-amplification and qRT-PCR analysis: First-strand cDNA was synthesized using 150 ng of total RNA for enriched CEC samples and 1 ug of total RNA for whole blood samples as well as High-Capacity cDNA Archive kit (Applied Biosystems, Foster City, CA). The cDNA was amplified with the ABI TaqMan PreAmp method (Applied Biosystems) and reagents according to the manufacturer's instructions. The selected candidate genes and the housekeeping control gene (GAPDH) were evaluated using the qRT-PCR assay with the pre-amplified material. PCR amplification was performed on the Bio-Rad real-time PCR Detection system (Life Science Research, Hercules, CA) using the 96-well block format with a 25-µl reaction volume. The concentration of the primers and the probes was 9 and 2.5 µmol/l, respectively. The reaction mixture was incubated at 95˚C for ten minutes to activate AmpliTaq®, followed by 40 cycles at 95˚C for 15 sec for denaturing and at 60˚C for 45 sec for annealing and extension. All TaqMan® Assay primer and probe sets were purchased from Applied Biosystems sequences of which are available by request. PCR data of Ct values were exported for further analysis. The results were considered valid when the Ct value of GAPDH was ≤30 for enriched

CEC samples and ≤20 for whole blood samples as well as when no template control had undetectable Ct. By using this threshold, five of the 60 enriched CEC RNA samples (8.3%) and three of the 76 whole blood RNA samples (4%) were excluded from further analysis (GAPDH cut off = mean Ct ± 2SD). ΔCts normalized by GAPDH were applied for all data analysis. An elastic net model was trained using the qPCR data to predict acute myocardial infarction [3]. Parameters for the elastic net were: alpha of 0.5, binomial family, a logistic link function and a lower limit of zero. The performance of the model was evaluated using leave-one-out cross validation and the receiver-operator characteristic curve.

References:

1. Sonnenberg, A. *et al.* Rapid electrokinetic isolation of cancer-related circulating cell-free DNA directly from blood. *Clin. Chem.* **60,** 500–509 (2014).
2. Irizarry, R. A. *et al.* Exploration, normalization, and summaries of high density oligonucleotide array probe level data. *Biostatistics* **4,** 249–64 (2003).
3. Friedman, J., Hastie, T. & Tibshirani, R. Regularization Paths for Generalized Linear Models via Coordinate Descent. *J. Stat. Softw.* **33,** 1–22 (2010).
4. Robin, X. *et al.* pROC: an open-source package for R and S+ to analyze and compare ROC curves. *BMC Bioinformatics* **12,** 77 (2011).
5. Smyth, G. in *Bioinforma. Comput. Biol. Solut. Using R Bioconductor* (Gentleman, R., Carey, V., Huber, W., Irizarry, R. & Dudoit, S.) 397–420 (Springer-Verlag, 2005).
6. Subramanian, A. *et al.* Gene set enrichment analysis: a knowledge-based approach for interpreting genome-wide expression profiles. *Proc. Natl. Acad. Sci. U. S. A.* **102,** 15545–50 (2005).
